# Mr. Swan's Enquiry into the Action of Mercury on the Living Body

**Published:** 1823-09-01

**Authors:** 


*An Enquiry into the Action of Mercury on the Living Body.* By JOSEPH SWAN, Member of the Royal College of Surgeons, and Surgeon to the Lincoln County Hospital. 8vo. sewed, pp. 30, London, 1822.

MR. SWAN believes that there is no active medicine, the employment of which is more general, or its operations less understood, than mercury. This may be true, for there are few, or perhaps no medicines of such extensive and varied influence on the human frame as mercury in its different preparations. What organ almost, in the body, is there, whose function may not be acted on by mercury, either alone or combined with some other medicine? The stomach, intestines, liver, pancreas, and, in fact, all the glands of the body, come under its influence when introduced either by the mouth or skin. It is to be acknowledged, however, that when injudiciously employed, and in some constitutions under the most cautious management, changes are produced inimical to future health—and these changes are deserving of the most serious investigation.

Our author seems to be in doubt whether the operation of mercury on the human frame is to be accounted for on the principle of absorption and accumulation in the body, or by an increased action produced in some other way than by absorption. The peculiar taste produced in the mouth, and the change in silver worn about the body seem to favour the doctrine of absorption; but these circumstances, Mr. Swan thinks, are fallacious, as he has known the mercurial taste exist where no mercury had been used—and he seems to doubt the occurrence of the change in silver when the mineral has only been used by the mouth.

"When mercury is taken into the body in only a small quantity, it proves a gentle stimulant to the secretory and absorbent systems, and causes the functions of them and the viscera to be performed in a more perfect manner, and no disturbance of the system is the consequence. But when it is taken in continued doses, it excites salivation or an increase of other discharges, as urine; and not only does this, but likewise frequently produces a furred tongue and much irritative fever. In some, the nervous system is much affected, debility and lowness of spirits are produced, and even after the mercury is discontinued, the system is very long in recovering." 7.

Mr. Swan conceives that mercury acts upon the villous coat of the stomach and intestines by producing a peculiar irritation, "for it is not the quantity used which excites salivation and the usual consequences of mercury, but the irritating form in which it is administered." When, therefore, he observes the effects produced by mercury, and finds that it cannot be detected in the blood or in any of the secretions, he cannot help supposing that it acts on the nervous system, and most particularly on that part of it formed by the grand sympathetic nerve. This, he thinks, is proved by the following case.

"On dissecting the grand sympathetic nerve, I found its ganglia and branches larger than I ever observed them before; I also observed an increased size of the par vagum. I could not satisfy myself the other nerves were enlarged. The subject had an encysted tumour in the liver about the size of a pigeon's egg, and the substance of this viscus when divided was streaked with red, in a peculiar manner, which appearance seemed to me the effect of inflammation.

"I supposed the system had been under the influence of mercury, for both sides of the face were much swoln; the salivary and absorbent glands were much enlarged; the teeth were loose, and there was a separation of the gums.

"On opening the spinal canal, I found much glairy fluid on the surface of the dura mater; and I never observed its existence in the same degree in any other subject. The body was emaciated, but as far as I could judge, death had taken place rather suddenly, for the emaciation was not in the greatest degree. I did not observe disease in any of the other viscera. The brain was firm and sound, but the medulla spinalis was very soft.

"On finding this state of the grand sympathetic nerve, and par vagum, I was led to suppose, that it was, as I have before stated, the effect of mercury; and it being allowed that this medicine cannot be traced either in the blood or any of the secretions, and that its effects increase in proportion to the quantity used, if it be not accumulated in the system, as I do not conceive to be the fact, we must necessarily imagine that a change is produced in some very important part by it: and this change, from considering the symptoms produced by mercury, I cannot help concluding is in the nervous system, and that the enlargement of the nerves in this instance was the effect of the mercury. I could not learn the history of the case." 16.

As Mr. Swan must have been aware that little could be concluded from an insulated case of this kind, he instituted some experiments on dogs, with the view of ascertaining whether the nerves really became affected by the use of mercury. These experiments, however, were only two in number, and to us appear far from satisfactory. We shall give the results. The dogs took calomel and sometimes opium, generally night and morning, in moderate doses (from two to five grains) till ulcers were produced in the mouth, till great constitutional derangement took place—and, in short, till death was occasioned by the mineral. In the first experiment, besides some ulcers in the mouth, and loose teeth, there were reddish spots on the villous coat of the small intestines, and marks of inflammation in the mucous membrane of the large. "The ganglia of the grand sympathetic nerves were very vascular, and likewise the par vagum; and all the nerves were more vascular than in a state of health. On one of the sciatic nerves there was a spot of ecchymosis." In the second experiment the appearances were nearly the same. The par vagum was inflamed, especially at its superior part, and again where it communicates with the inferior cervical ganglion. All the ganglia of the great sympathetic nerves were inflamed, and likewise portions of the nerves themselves. The sciatic nerves were also more vascular than usual, especially near the ischiatic notch. Mr. Swan examined the ganglia of several of the spinal nerves 50 or 60 hours after death, and still found them so full of vessels as to leave no doubt in his mind that they had partaken of the inflammatory action.

"The results of these experiments lead me to believe that mercury produces first an irritation in the grand sympathetic nerve, communicated to it from the termination of its branches on the villous coat of the intestines; and if its use is persevered in, inflammation is soon the consequence; whilst, at the same time, the same irritation is communicated to the other nerves. But it may be necessary to inquire why the nerves of the limbs are not found enlarged always, as well as those of the grand sympathetic nerve. I conceive that mercury acts more decidedly on the grand sympathetic than on any of the other nerves, and that it is only after a length of time that these other nerves become enlarged. In the second experiment, the par vagum, the nerves forming the axillary plexus, and those of the sciatic nerve were very red from vessels near their communication with the grand sympathetic nerves, and the diminished vascularity was very evident the further they were removed from these. I therefore think it probable that the enlargement of the grand sympathetic nerve in the dissection I have mentioned, without any enlargement of the other nerves except the par vagum, is what usually takes place, and that the other nerves become enlarged only where the use of mercury is very long continued. The increased vascularity of all the nerves will, I think, account for the pains of the limbs and frequently for their weakness. Will not the decided effect produced on the par vagum, likewise account particularly for the aggravation or the coming on of consumptive symptoms where there was a predisposition to that disease, by a long course of mercury, and explain why a few doses of mercury, given where the symptoms are those of consumption, and depending only on a disordered state of the chylopoietic viscera, will remove such disorder?

"If mercury affects the grand sympathetic nerves, it may then fairly be asked, how its influence is communicated when rubbed on the skin? I conceive it probable that it may irritate the nerves of this part, and that this irritation may spread from its nerves to the rest of the nervous system; but whether the influence of mercury is communicated by the nerves of the skin to the other parts of the system, or in any other manner, I am firmly persuaded that it is exercised almost entirely on the nervous system." 25.

We agree with our author that, as mercury appears to affect the nervous system (a fact which has long been known without experiments on the subject) it is necessary not to be profuse in its exhibition in ordinary cases. He observes that—

"It is generally believed that when the stools are black, or have not the natural appearance, mercury will so alter the secretions of the viscera as to produce a healthy appearance of them; and though it has this desired effect when properly administered, yet it sometimes happens that, instead of the secretions becoming better, they are more and more discoloured, and in such cases it is supposed necessary to push on its use with redoubled diligence; but as, in some of these instances, the parts are already too irritable, the more this state is increased by the mercury, the more discoloured the evacuations become, whilst after some time, when it has been discontinued, and quieting medicines used, the irritation will cease, and the secretions become natural.

"When mercury is given for the cure of an ulcer which depends upon an improper state of the digestive organs, a few doses will change the secretions of these organs to a healthy state, and the sore will immediately begin to heal, but if the cure depends on a change to be produced on the ulcer by the mercury, it must be effected either through the system or some local application. If through the system, how is the change produced? Mercury is not in the system, but is applied to parts whose nerves are susceptible of its influence. Through these the whole nervous system is affected, and the change of action in the termination of the nerves produces a corresponding change in the minute arteries; as constitutions vary, so when this action is carried beyond a moderate degree in a weak or irritable one, the part is stimulated beyond its powers, and sloughing or death are produced." 27.

Our author further observes that, when for a supposed syphilitic ulcer mercury is given, it so changes the sore as to produce a healthy state. But if the mercury be continued longer (he should have said "too long") the ulcer becomes (he should have said "sometimes") irritable, and may frequently be cured by leaving off the medicine, and giving something to reduce irritability.

"When the ulcer has begun to heal, the restorative process often stops, or the ulcer spreads; the parts have become languid from the previous too great action, and then if mercury is given again, it again stimulates the nerves, and the parts again dispose for a time for healing; again perhaps they become too irritable and spread or do not mend, and then by leaving off mercury and giving quieting medicines the ulcer again begins to assume a healthier appearance; and this varied state continues to teaze the patient for a great length of time, till at last by the frequent use of mercury, the actions of the whole body become changed, and fresh symptoms shew themselves, and in this manner I conceive diseases resembling syphilis are frequently produced." 28.

We have now given the sum and substance of the pamphlet, and we think that the insulated case in the human subject goes for little, while the experiments on the two dogs go for still less. The whole, we imagine, only supports the rational opinion now pretty generally entertained, that the *use* of mercury is a great good in the practice of physic, while the abuse of it is a crying evil.

